# Determining the Mouth-to-Microphone Distance in Rigid Laryngoscopy: A Simple Solution Based on the Newly Measured Values of the Depth of Endoscope Insertion into the Mouth

**DOI:** 10.3390/jcm12247560

**Published:** 2023-12-07

**Authors:** Dominika Valášková, Jitka Vydrová, Jan G. Švec

**Affiliations:** 1 Voice Research Lab, Department of Experimental Physics, Faculty of Sciences, Palacký University, 779 00 Olomouc, Czech Republic; dominika.valaskova01@upol.cz; 2 Voice and Hearing Centre Prague, Medical Healthcom, Ltd., 110 00 Prague, Czech Republic

**Keywords:** rigid laryngoscopy, oral cavity length, mouth-to-microphone distance, voice recording

## Abstract

Mouth-to-microphone (MTM) distance is important when measuring the sound of voice. However, determining the MTM distance for laryngoscope-mounted microphones during laryngoscopic examinations is cumbersome. We introduce a novel solution for such cases, using the depth of insertion of the laryngoscope into the mouth *D*_I_ as a reference distance. We measured the average insertion depth, *D*_I_, in 60 adult women and 60 adult men for rigid laryngoscopes with 70° and 90° view. We found the *D*_I_ for the 70°/90° laryngoscope to be 9.7 ± 0.9/9.4 ± 0.6 cm in men, 8.9 ± 0.9/8.7 ± 0.7 cm in women, and 9.3 ± 0.9/9.0 ± 0.7 cm in all adults. Using these values, we show that, for microphones fixed at 15–40 cm from the tip of the laryngoscope, the final MTM distances are between 5 and 35 cm from the lips, and the standard uncertainties of these distances are between 16% and 2.5%. Our solution allows laryngologists and laryngoscope manufacturers to set and estimate the MTM distance for any rigid laryngeal endoscope with a microphone attached with reasonable accuracy, avoiding the need to measure this distance in vivo in routine practice.

## 1. Introduction

**Figure 1:**
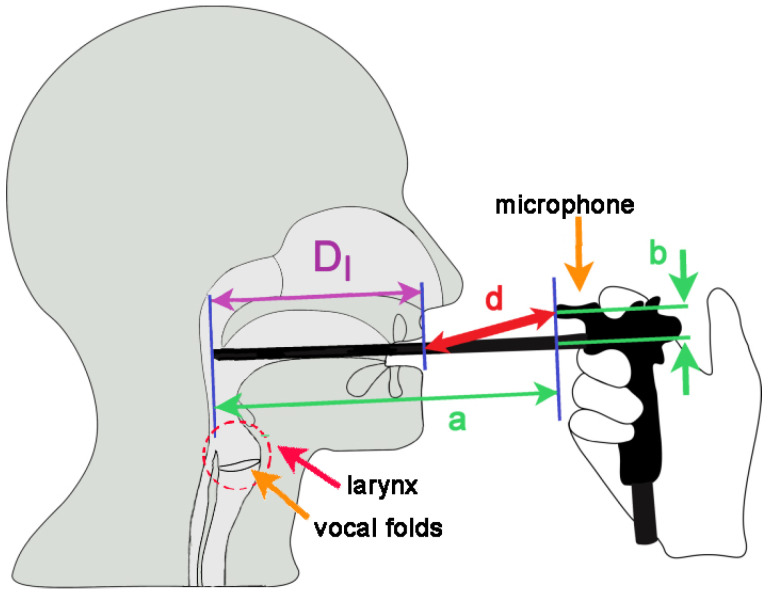
Insertion of a rigid laryngeal endoscope (laryngoscope) into the mouth to observe the larynx and the vocal folds during voice production in clinical voice examination. A small microphone attached to the laryngoscope captures the produced voice. The mouth-to-microphone (MTM) distance, *d*, influences the properties of the captured sound. This distance depends on the position of the microphone with respect to the tip of the laryngoscope (parameters *a* and *b*) and the depth of insertion of the laryngoscope into the mouth, *D*_I_.

Laryngeal endoscopy (laryngoscopy) is the basic tool for the clinical examination of the voice. State-of-the-art laryngoscopy utilizes rigid and flexible laryngeal endoscopes (laryngoscopes) that are inserted into the mouth or nose, respectively, in order to visualize the larynx and the vocal folds (Figure 1). Here, we focus on the rigid endoscopes that allow for different types of endoscopic video cameras to be attached to capture laryngeal motion during phonation. Three basic laryngeal endoscopic techniques are used in laryngology for this purpose: laryngeal stroboscopy, high-speed videoendoscopy, and videokymography [1,2,3,4,5]. These methods visualize the larynx by using the same laryngoscopic approach but utilize three different types of video cameras, providing different types of laryngeal videos. All mentioned techniques aim at observing the vocal fold vibrations, revealing possible abnormalities of the vocal fold function. 

Modern laryngoscopic devices capture the sound by a small microphone attached directly to the laryngoscope or to a camera head to relate the vocal fold abnormalities to the produced vocal sound. The captured sound is used to determine basic vocal characteristics, e.g., the vocal fold vibrational frequency (*f*_o_) and sound pressure level (SPL), which can be displayed on a monitor [6]. This is relevant because the laryngeal adjustment and vocal fold behavior change with the *f*_o_ and SPL.

In order to interpret the recorded sound correctly, it is important to know the distance of the microphone from the mouth [7,8]. Wendler recommended setting this distance to 15 cm during laryngoscopy [9]; however, this recommendation has not been universally adopted. When the microphone is attached to the laryngoscope, or to the camera head mounted on the laryngoscope, the mouth-to-microphone (MTM) distance, *d*, cannot be set directly, because it depends on the depth of insertion of the laryngoscope into the mouth *D*_I_ (Figure 1). Therefore, to determine the MTM distance, *d*, we need to measure it directly during the laryngoscopic procedure. This measurement normally requires three people to be involved (the examiner performing the laryngoscopy, the examiner performing the distance measurement, and the subject undergoing the rigid laryngoscopy). Such a measurement is doable but cumbersome and time-consuming to perform in clinical practice for every examined client.

In this paper, we introduce an alternative method of determining the MTM distance for microphones attached to rigid laryngoscopes or to camera heads mounted on rigid laryngoscopes. Our method is based on knowledge of one key parameter—the depth of insertion of the laryngoscope into the mouth, *D*_I_. As shown in Figure 1, the MTM distance, *d*, can be determined by subtracting the *D*_I_ value from the microphone position with respect to the tip of the laryngoscope. If the laryngoscope insertion depth, *D*_I_, is reasonably similar across adult males and across adult females, the MTM distance does not have to be measured for each patient but can rather be universally specified for each laryngoscope, using a simple formula. However, to the best of our knowledge, so far, there is no reliable information on how deep the endoscope is placed into the mouth when performing rigid laryngoscopy and how the variability changes across subjects. Therefore, the first aim of this study is to determine the average insertion depth and its variation in adults for two common types of rigid laryngoscopes used in clinical practice—those with angles of view of 70 and 90 degrees [10,11]. Our specific questions and hypotheses related to the measurements of the laryngoscope insertion depth, *D*_I_, were as follows:

1. Is the insertion depth different between male and female patients? We hypothesized the insertion depth to be larger in males, due to their longer vocal tract.
2. Is the insertion depth different for the laryngoscopes with a 70° and 90° view? We hypothesized the insertion depth to be larger in the 70° laryngoscope type since its tip is expected to be placed lower in the oropharynx, closer to the larynx.

The second aim of this study is to formulate empirical rules for determining the universal laryngoscopic MTM distance, *d*, in adults for the 70° and 90° rigid laryngoscopes with an attached microphone. To determine the possible difference in the MTM distance, *d*, estimated by the rules from its real value, the standard uncertainty is specified for adult males, adult females, and for all adults from the measured variability of the laryngoscope insertion depth, *D*_I_, across subjects.

## 2. Materials and Methods

**Figure 2:**
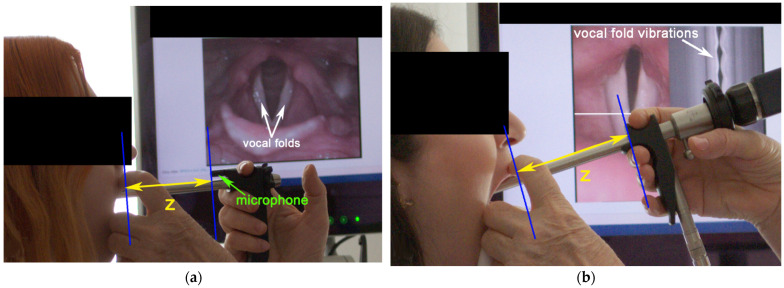
Side images obtained during (**a**) laryngeal stroboscopy (90° laryngoscope with an embedded microphone, L90S) and (**b**) videokymography (70° laryngoscope, L70K). The outside distance, *z*, was measured to derive the laryngoscope insertion depth into the mouth. Notice the monitor in the background showing the laryngeal images with the vocal folds—these were used for checking the correct placement of the laryngoscope in the mouth.

This is a cross-sectional study of 120 adult subjects. Three rigid laryngoscopes with 90° and 70° angles of view were used for the study. The measurements were performed from side photographs of the subjects taken during laryngoscopy. The length of the laryngoscope tube visible outside the mouth, *z* (Figure 2), was subtracted from the known full length of the laryngoscope, *l*, to derive the laryngoscope insertion depth, *D*_I_. The details of the materials and methods are given below.

### 2.1. Subjects

One hundred twenty human adults (sixty male, age 50 ± 18, age range 19–91; sixty female, age 45 ± 18, age range 18–89) served as subjects for our study. The subjects were prospectively recruited from patients visiting a laryngologist at the clinical institute to which the second and last author belong. They were White Czech patients, except for one Hispanic patient. They visited the clinic for an outpatient laryngeal examination for various voice disorders or prevention purposes. The specific laryngeal diagnoses were of no significance for this study; we were interested only in measuring the depth of laryngoscope insertion into the mouth during the examinations. None of the subjects had the laryngeal skeleton deformed due to an accident or removed due to cancer. Only those subjects in which the laryngeal exam could be successfully completed and who gave their consent to the examination were included in the study. The patients with an obstructed view or no view of the vocal folds during phonation were excluded.

### 2.2. Examination Procedures and Division of the Subjects into Groups

The subjects were photographed from the side while undergoing a routine laryngoscopic examination procedure. All the subjects gave their consent for the photography. The Ethics Board of the clinical institute that the second and last authors belong to approved of the consent form and the study. Apart from taking the photographs, no special investigations were performed for the purpose of this study. The patients were seated comfortably in the examination chair and instructed to sustain a phonation of vowel/e/ or syllable/he/ at a comfortable pitch and loudness. Both the insertion of rigid laryngoscopes and the examination process were performed by a laryngologist with 30 years of clinical experience. 

In the beginning, each participant underwent a laryngeal stroboscopic examination. This was performed using a 90° view laryngoscope (further labeled L90S), which is commonly used in Europe. The participants were then divided into three equally sized groups, each containing 40 subjects (20 females and 20 males). The division was based on the need for an additional laryngeal videokymographic examination, which was performed to obtain more accurate information on vocal fold vibrations, thus allowing for a more detailed diagnosis when needed. Group 1 underwent laryngeal videokymographic examination using the 70°-view laryngoscope (L70K). Group 2 underwent laryngeal videokymographic examination using the 90°-view laryngoscope (L90K). The division of the subjects into Groups 1 and 2 was random. Subjects from Group 3 were not indicated for any additional laryngeal examination procedure. The distribution of the subjects into the groups is summarized in Table 1. The ANOVA test revealed no statistically significant difference in age among the subgroups (ANOVA: p_age-females_ = 0.397; p_age-males_ = 0.590).

### 2.3. Examination Equipment

For the stroboscopic examination, we used the endoscopic system Xion EndoSTROB together with a L90S laryngoscope. This was a 90° rigid endoscope containing an integrated camera and microphone (Xion zoom laryngoscope type 130 310 629, Ø 10 mm, with handle and integrated light guide cable). For the subsequent VKG examination, we used the 2nd-generation videokymographic camera (Cymo 2156) and the 300 W endoscopic xenon light source (FX 300 A, Fentex Medical, Neuhausen ob Eck, Germany). The camera was alternately attached to one of two different rigid laryngoscopes for the VKG examinations. For Group 1, we used the L70K laryngoscope, i.e., the 70° Xion zoom laryngoscope (type 130 3210 527, Ø 10 mm). For Group 2, we used the L90K laryngoscope, i.e., the 90° Xion zoom laryngoscope (type 130 310 529, Ø 10 mm). The laryngoscopes were attached to the VKG camera head, using a C-mount objective adapter (R. Wolf, Knittlingen, Germany, type 8523.272, 27 mm focal length). The only reason for using the two different laryngoscopes for videokymography was the interest in finding whether there is a difference in the depth of laryngoscope insertion into the mouth between the 70° and 90° laryngoscope types since these two laryngoscope types are commonly used at different laryngology departments around the world [10,11].

Photographs were taken from the client’s side, using the Nikon D3100 camera with a 4608 × 3072 pixel resolution. The photographic view was focused on the client’s head and included the hand of the examiner holding the laryngoscope inserted into the mouth. A computer monitor displaying the laryngoscopic view was intentionally placed in the background (Figure 2) and was utilized to check whether the laryngoscope was fully inserted into the mouth, providing the full view of the vocal folds (Figure 2a). In the photographs taken during the VKG examination, the display monitor had to show the VKG line being placed perpendicularly to the glottis, as well as visible vocal fold vibrations in the videokymograms (Figure 2b). Multiple photographs were taken during every single examination. In total, 962 photographs (464 photos of 60 females; 498 photos of 60 males) were taken. For each subject, the age was noted.

### 2.4. Calibration Measurements—Laryngoscope Lengths

**Figure 3:**
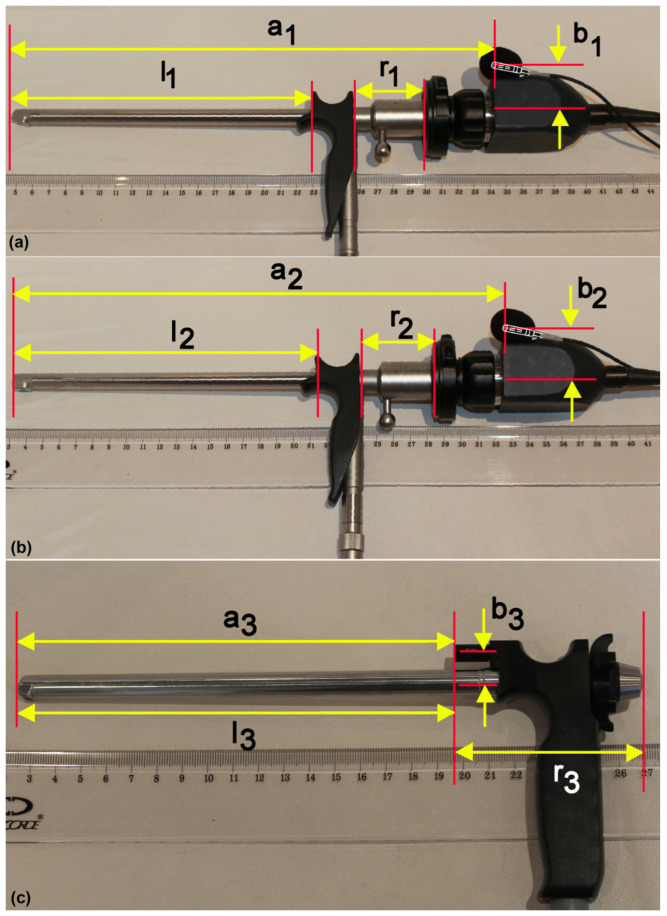
Examples of calibration photographs of the three rigid laryngoscopes used in this study: (**a**) L70K, (**b**) L90K, and (**c**) L90S. The lengths of the tubes, *l*_1_, *l*_2_, *l*_3_, and of the reference parts, *r*_1_, *r*_2_, *r*_3_, as well as the horizontal and vertical positions of the microphone from the tip of the laryngoscope (distances *a*_1_, *a*_2_, *a*_3_, and *b*_1_, *b*_2_, *b*_3_, respectively), were determined for the three laryngoscopes, respectively, using these photographs. The results are listed in Table 2. In (**a**,**b**), the microphone is fixed to the VKG camera head and covered by a protective acoustic foam; the location of the microphone inside the foam is indicated by the drawing. In (**c**), the microphone is embedded directly in the laryngoscope L90S.

Multiple photographs of the rigid laryngoscopes used for this study were taken for reference purposes to find the length of the tubes of the used laryngoscopes. In total, 20 photos were taken (L70K: 8 photos; L90K: 7 photos; L90S: 5 photos) and used for calibration measurements of the lengths. The laryngoscopes were laid on a white background next to the ruler, giving clearly visible marks of lengths (Figure 3). The ruler was used to calibrate the photograph measurements in millimeters. The length of the tube, *l*, and the reference part, *r*, was determined from the photographs, as well as the distances *a* and *b* defining the microphone’s position with respect to the tip of the laryngoscope, and the distance *c* of the center of the objective lens from the tip of the laryngoscope (Table 2). The calibration and the measurements were performed using the freeware program ImageJ.

### 2.5. Measurements of the Depth of Laryngoscope Insertion into the Mouth

In order to find out the depth of the laryngoscope insertion into the mouth, we measured the length of the tube remaining outside the mouth during laryngoscopy from the photographic images (Figure 2, distance *z*). To find this distance in centimeters, we used the known size of the reference part, *r*, of each laryngoscope (Table 2 and Figure 3). The end of the mouth was determined by drawing a line touching the upper lip that was perpendicular to the laryngoscopic tube for each patient (solid blue line touching lips, Figure 2). The depth of the laryngoscope insertion into the mouth, *D*_I_ (Table 3), was obtained by subtracting the measured outside length, *z*, from the known total length, *l*, of the laryngoscope tube (Table 2). 

The statistical analysis was performed using the programs MS Excel and Statistica. The one-sample Kolmogorov–Smirnov test (K-S test) was used for the results from each laryngoscope to find whether the data were normally distributed. To compare the different groups (sex or laryngoscope type), we first performed the test of equality of variances: the F-test with the null hypothesis of two groups having the same variances. The F-test was a prerequisite for the choice of the subsequent two-sample *t*-test with equality or inequality variances and paired two-sample *t*-test for means. To determine significance, we used α values of 0.05, 0.01, and 0.001. To counteract the problem of multiple comparisons during the K-S test and *t*-tests, we used the Holm method [12]. For the Holm method, we sorted all *p*-values from the lowest to highest (*p*_1_, *p*_2_…*p*_i_…*p*_K_) and defined corrected alpha levels as follows:

(1) $$\alpha^{*}=\frac{\alpha }{K-i+1}$$

where K is the number of *p*-values, and i is the order of *p*_i_-values. Every *p*-value had a different α* value to be compared with. A more detailed explanation of the measuring steps can be found in [13,14].

## 3. Results

**Figure 4:**
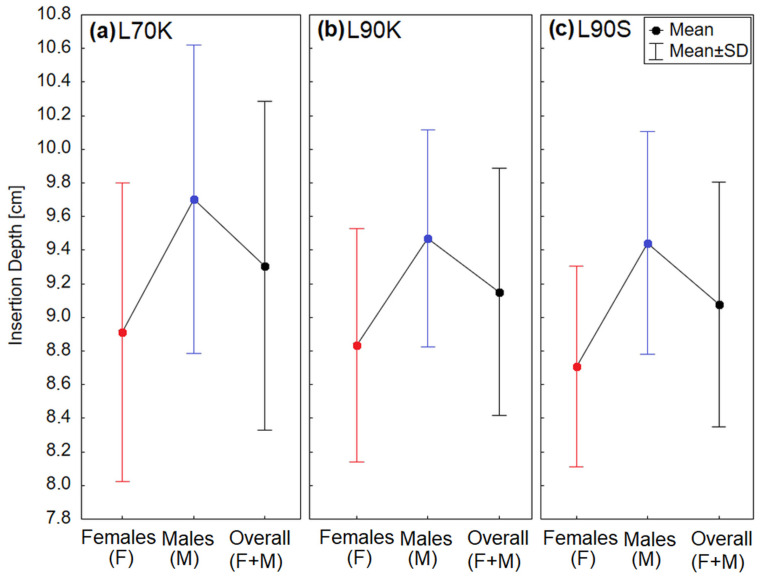
The insertion depth, *D*_I_, of different types of laryngoscopes for females (red), males (blue), and all adults (females plus males, black): (**a**) laryngoscope L70K, (**b**) laryngoscope L90K, and (**c**) laryngoscope L90S.

Figure 4 shows the average insertion depth in females and males, as well as in all adults (i.e., females pooled with males), measured for the three different types of rigid laryngoscopes, L70K, L90K, and L90S. The numerical results are provided in Table 3. The K-S test proved that all data were normally distributed (K-S test: $\alpha_{0.05}^{*}$ = 0.006–0.05, *p* = 0.06–0.6 for the nine subgroups displayed in Figure 4).

### 3.1. Female–Male Differences

The results of the measurements comparing the laryngoscope insertion depths in female and male subjects are provided at the bottom of Table 3. The *t*-test (two-sample test with equal variances) showed that the insertion depth significantly differed between males and females, which was true for all three laryngoscopes used. On average, the insertion depth was 7 mm larger in male than in female subjects (see also Figure 4). The F-test revealed no significant differences in variances of the insertion depth between females and males for any of the laryngoscopes.

### 3.2. Differences among the Laryngoscopes

Since each subject in Groups 1 and 2 was examined using two different laryngoscopes (L70K and L90S for Group 1; L90K and L90S for Group 2), we could investigate the paired differences in the insertion depth of the laryngoscopes. The results are provided in Table 4. The 70° laryngoscope (L70K) was inserted, on average, about 3 mm deeper into the mouth than the 90° laryngoscope (L90S). The paired *t*-test revealed that this difference fulfilled the significance criterion for females (*p*-value of 0.04), but not for males and the adults (males and females pooled together). Much smaller, insignificant differences of 0.4–0.5 mm were found between the average insertion depths of the two 90° laryngoscopes (L90S and L90K, used for stroboscopy and videokymography, respectively). Hence, in the last column of Table 3, we provide the pooled measurement results for both of the 90° laryngoscopes.

The variability of the insertion depth among the subjects was also found to be slightly larger for the 70° than for the 90° laryngoscope (standard deviations of 9 mm versus 6–7 mm, respectively, as shown in Table 3 and by the whiskers in Figure 4). This difference passed the threshold of statistical significance in females and in all adults, as indicated by the *p*-value 0.04 obtained from the F-test (see Table 4).

## 4. Discussion

The obtained results confirmed our two hypotheses to be true:

1. The laryngoscope insertion depth was larger in male than female patients;
2. The insertion depth was slightly larger for the 70°-view laryngoscope than for the 90°-view laryngoscope.

The specific results and their implications are discussed below.

### 4.1. Female–Male Differences in Insertion Depth

Our results show that the laryngoscope is inserted deeper in the mouth in males than in females (Table 3). The difference was about 7 mm, and it was found statistically highly significant. This could be related to the significant anatomical differences in the vocal tract length between females and males observed by other authors, with the female vocal tract being shorter [15,16,17,18,19,20]. It is worth comparing our results specifically to the anatomical measurements of the horizontal length of the vocal tract or of the oral cavity depth reported in the literature. Vorperian [17,18,19] defined the horizontal length of the vocal tract as the distance from a line tangential to the lips to the posterior pharyngeal wall. Their data reveal these lengths to be 9.26 ± 0.63 cm for females and 9.81 ± 0.96 cm for males. Similar results were found by Goldstein [20], who reported the oral cavity length plus lips to be 9.2 cm for females and 9.7 cm for males. These lengths are only a few millimeters longer than our values for laryngoscope insertion depth provided in Table 3, which is well understandable: when inserted, the tip of the laryngoscope typically does not touch the posterior pharyngeal wall to avoid a gagging reflex. The distance of the scope’s tip from the pharyngeal wall may thus account for the differences between our measurements and those of Goldstein [20] and Vorperian et al. [17,18,19]. The female-to-male differences of 5–6 mm found by Goldstein and Vorperian et al. are close to the 7 mm differences found in our data.

### 4.2. Insertion Depth Differences among Different Laryngoscopes

Regarding the differences among the laryngoscope types, the results indicate the following:

1. The 70° scope (L70K) was inserted about 3 mm deeper into the mouth, and its insertion depth varied slightly more among the subjects than in the case of the 90° laryngoscope (L90S);
2. The two 90° laryngoscopes (L90S and L90K) could be considered similar in terms of their insertion depth (Table 4).

Although not passing the significance threshold in all cases, the 3 mm larger insertion depth for the 70° laryngoscope type is not surprising given the fact that its construction allows the tip of the laryngoscope to be placed in the oro-pharyngeal cavity slightly lower and closer to the larynx than the 90° laryngoscope [10]. On the other hand, there was a negligible difference of less than 0.5 mm between the two 90° laryngoscopes used here. Interestingly, the L90K and L90S laryngoscopes were of highly different construction (classical versus chip-on-the-tip design), and they had significantly different positions of the centers of their objective lenses from the tip of the laryngoscope: 6.1 ± 0.1 mm versus 3.0 ± 0.1 mm, respectively (recall Table 2). The more distant position of the lens from the tip was not found to be compensated by deeper insertion of the laryngoscope into the mouth—the 3 mm difference in the lens positions did not appear to have any significant influence on the laryngoscope insertion depth in our study. This suggests that the depth of laryngoscope insertion into the mouth is similar for rigid laryngoscopes of different constructions when they have the same angle of view. Based on these results, we assume the insertion depth to be similar for rigid laryngoscopes of the same type within and among different manufacturers. Verifying this assumption, however, would require a more extensive study with several more laryngoscopes, which exceeded our current possibilities. Nevertheless, considering the ca. ±7 mm variation in the insertion depth found among different subjects, as indicated in Table 3, and only the 0.5 mm difference between the two 90° laryngoscopes, as indicated in Table 4, we could safely assume that the insertion depth differences among different laryngoscopes of the same type are smaller than the depth variation among different subjects.

### 4.3. Stroboscopy–Videokymography Differences in Insertion Depth

Our study showed negligible differences in the laryngoscope insertion depth between stroboscopy and videokymography (Table 4; *p*-values above 0.55 for the *t*-test and above 0.32 for the F-test, columns L90S vs. L90K). We do not find this surprising: the videokymography camera provides a dual image—besides the videokymographic view, there is simultaneously also a full laryngeal view (recall Figure 2b), which is no different from the views provided by the cameras used for laryngeal stroboscopy. This full laryngeal view is used as a basis for adequate laryngoscope insertion. Furthermore, the stroboscopic laryngeal view is identical to the laryngeal view obtained in high-speed laryngeal imaging; the only difference is the number of frames per second (fps) delivered by the video cameras (50–60 fps in stroboscopy versus 2000 fps or more in high-speed imaging). Therefore, there is no principal difference between stroboscopy and the other laryngoscopic methods (videokymography and high-speed videolaryngoscopy) concerning the insertion of the laryngoscope into the mouth. We were able to test the stroboscopy—videokymography differences only with the 90° laryngoscope; nevertheless, we find no logical reasons to assume that the results for the 70° laryngoscope should be different. Hence, we consider the insertion depths measured in stroboscopy to be representative also for videokymography and high-speed videoendoscopy, regardless of the type of rigid laryngoscope used. However, the fact that the stroboscopic device allowed us to use only the camera-integrated 90° laryngoscope and not the 70° laryngoscope is a limitation of this study.

### 4.4. Rules for Estimating the MTM Distance in Rigid Laryngoscopy

The empirical data on the insertion depth, *D*_I_, can be used to estimate the MTM distance, *d*, for the laryngoscope-attached microphones. To obtain this distance, it is only necessary to know the horizontal and vertical placement of the microphone with respect to the tip of the laryngoscope (distances *a* and *b*, respectively, as indicated in Figure 1 and Figure 3). We measured these distances by using calibrated photographs, but, in principle, they can also be measured directly, using a simple ruler. Once the *a* and *b* values are known, the empirical values of *D*_I_ listed in Table 3 can be used, and the MTM distance, *d*, can simply be determined using the Pythagorean theorem, according to the formula

(2) $$d=\sqrt{{\left({a-D}_{I}\right)}^{2}{+b}^{2}}.$$

**Figure 5:**
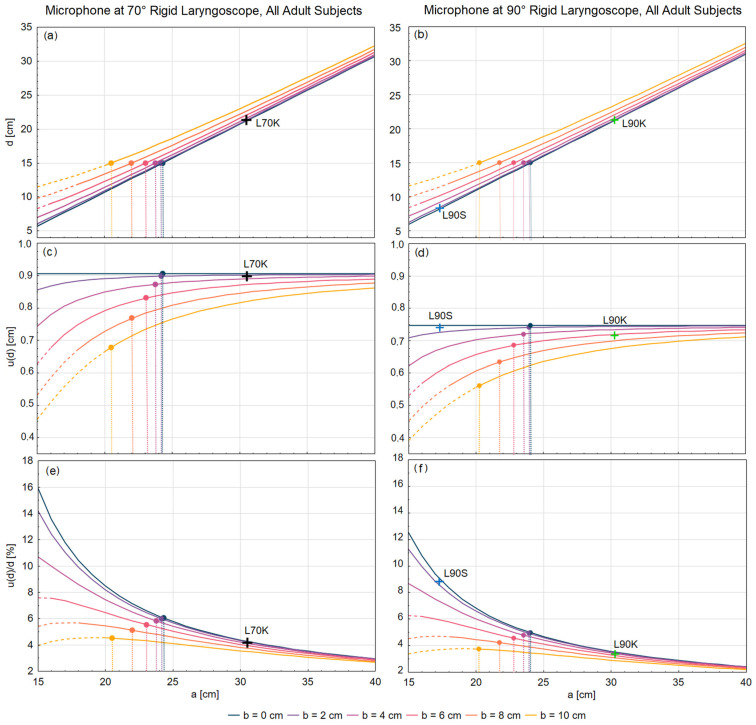
The average MTM distances, *d*, in adults (males and females pooled together) and their uncertainties *u*(*d*) for different microphone positions with respect to the tip of the laryngoscope (distances *a* and *b* indicated in Figure 1 and Figure 3). The results for the 70° laryngoscope are on the left (panels **a**,**c**,**e**) and for the 90° laryngoscope on the right (panels **b**,**d**,**f**). (**a**,**b**) MTM distance, *d*; (**c**,**d**) uncertainty of MTM distance, *u*(*d*); and (**e**,**f**) relative MTM distance uncertainty, *u*(*d*)/*d*. Crosses indicate results for the specific locations of the microphones used in this study. Dots indicate the standard MTM distance of 15 cm recommended by Wendler [9] for different values of *a* and *b*. For the separate female and male results, see Figure A1 and Figure A2 in the Appendix A.

Figure 5a,b shows the average MTM distances, *d*, for the 70° and 90° rigid laryngoscopes in adults (males and females pooled together). These were obtained from Equation (2) for different microphone positions, as defined by the distances *a* and *b* from the tip of the laryngoscope. In clinical setups, the values of *a* can be expected to be in the range from ca. 15 cm (in the case when the microphone is attached directly or embedded in the laryngoscope with a short tube) up to ca. 40 cm (in cases of microphones fixed on camera heads attached to long-tubed laryngoscopes). Similarly, the values of *b* can be expected to be in the range from ca. 0.5 cm (in the case when the microphone is attached directly or embedded in the laryngoscope) up to ca. 10 cm (in cases when the microphone is fixed on the top of a bulky camera head). These microphone positions result in the final MTM distances, *d*, between 5 and 33 cm (Figure 5a,b). In 1992, Wendler recommended standardizing the MTM distance during laryngoscopy to 15 cm [9]. Our method allows us to find the specific values of *a* and *b* to obtain the average MTD distance of 15 cm; these values are indicated in Figure 5a,b by dots.

In principle, the distances *a* and *b* could be provided directly by the manufacturer. When not provided (the current state-of-the-art), the distances *a* and *b* can be measured with an uncertainty of 1 mm or smaller by an educated researcher or technician in the clinical institute, using, e.g., a simple ruler.

### 4.5. Uncertainty of the MTM Distance

Expressing the uncertainties of *D*_I_, *a*, and *b* by the symbols *u*(*D*_I_), *u*(*a*), and *u*(*b*), according to International Organization for Standardization [21], we can determine that the overall uncertainty of the MTM distance, *d*, i.e., the possible difference of the true value from the value estimated by the Equation (2), is equal to

(3) $$\;u(d)=\sqrt{{\left(\frac{\partial d}{\partial D_{I}}\right)}^{2}u{\left(D_{I}\right)}^{2}+{\left(\frac{\partial d}{\partial a}\right)}^{2}u{\left(a\right)}^{2}+{\left(\frac{\partial d}{\partial b}\right)}^{2}u{\left(b\right)}^{2}}\;=\sqrt{\frac{u{\left(D_{I}\right)}^{2}+u{\left(a\right)}^{2}+{\left(\frac{b}{{a-D}_{I}}\right)}^{2}u{\left(b\right)}^{2}}{1+{\left(\frac{b}{{a-D}_{I}}\right)}^{2}}}.$$

The scaling factor, ${\left(\frac{b}{{a-D}_{I}}\right)}^{2}$, in Equation (3) relates the perpendicular and longitudinal distances of the microphone from the mouth. In practice, this ratio is expected to be smaller than 1 in order to keep the vertical angle of the microphone from the mouth less than 45°. This gives less weight to the uncertainty *u*(*b*) compared to *u*(*D*_I_) and *u*(*a*) in the numerator of Equation (3). Furthermore, the measurement uncertainties of the microphone position on the endoscope, *u*(*a*), the *u*(*b*), which are expected to be up to ca. 1 mm, are about 7–9 times smaller than the uncertainty of the insertion depth, *u*(*D*_I_), resulting from the inter-subject variation (recall the 7–9 mm standard deviations of the insertion depth, Table 3). The overall uncertainty of the MTM distance, *u*(*d*), is therefore predominantly determined by the variation in *D*_I_ within and among the examined subjects, and the combined uncertainties, *u*(*a*) and *u*(*b*), contribute to the overall uncertainty by less than ca. 2%. When neglecting the uncertainties *u*(*a*) and *u*(*b*), Equation (3) can be simplified to the following form:

(4) $$\;u(d)=\frac{{u(D}_{I})}{\sqrt{1+{\left(\frac{b}{{a-D}_{I}}\right)}^{2}}}.$$

Notice that the factor ${\left(\frac{b}{{a-D}_{I}}\right)}^{2}$ could also be neglected when the vertical position, *b*, of the microphone is much smaller than the horizontal distance of the microphone from the mouth (*a* − *D*_I_). In that case, the uncertainty of the MTM distance, *u*(*d*), becomes approximately equal to the uncertainty of the endoscope insertion depth, *u*(*D*_I_). This can also be noticed in Figure 5c,d, which depicts the results of Equation (3) graphically. For *b* = 0, the uncertainty, *u*(*d*), equals 0.91/0.75 cm in the case of the 70°/90° laryngoscope regardless of the horizontal position of the microphone, *a*. These values are less than 2% different from the standard deviations of the measured 70°/90° laryngoscope insertion depth, i.e., 0.90/0.74 cm (recall Table 3), as expected.

Figure 5e,f show the relative uncertainty, $\frac{u(d)}{d}$, for the varying positions of the microphone with respect to the tip of the laryngoscope. We see that the relative uncertainty decreases with the increasing distance, *a*. For a given *a*, the largest relative uncertainty is found for the microphone vertically placed at the closest proximity to the laryngoscope tube (*b* = 0 cm). In this case, the relative uncertainty of the MTM distance ranges from ca. 16% at *a* = 15 cm down to ca. 2% at *a* = 40 cm.

### 4.6. MTM Distances for L70K, L90K, and L90S Laryngoscopes

Finally, Table 5 provides information on the resulting MTM distances, *d*, for the three laryngoscope-fixed microphones used in this study. Recall that, in the case of L90S, the microphone was embedded in the laryngoscope (Figure 3c), whereas, in the case of the L70K and L90K laryngoscopes, the microphone position was at the proximal top edge of the camera head (Figure 3a,b). These microphone placement differences cause substantial differences in the MTM distance during laryngoscopy (ca. 21–22 cm for L70K and L90K versus ca. 8–9 cm for L90S, as indicated in Table 5).

### 4.7. Representativeness of the Sample Size in Relation to the Broader Population and Study Limitations

Our data are based on investigations of adult clients visiting the clinical outpatient center in Prague. Out of these, 119 self-reported to be White and 1 self-reported to be Hispanic. Previous studies found some differences in the volume of the oral and pharyngeal cavities among subjects of different ethnicities and races, but no significant differences were found in the lengths of these cavities [22,23]. Since the length dimensions are most relevant for our study, based on these findings, our results may be assumed representative for adults regardless of ethnicity and race. Nevertheless, more studies are welcome to verify this assumption further, as well as to verify our measurements with more laryngoscopes.

Our results are limited to adult subjects only. The laryngoscope insertion depth is expected to be smaller and age-dependent in children since the volume and length of the oral cavity increases with age during childhood [17,18,19]. More research is therefore needed to find a universal formula to determine the MTM distance in children. Nevertheless, rigid laryngeal endoscopy is less preferred to be used in children, often being replaced by flexible naso-laryngeal endoscopy [24].

Our study applies to rigid laryngeal endoscopes only; the results cannot simply be transferred to flexible naso-laryngeal endoscopes that are sometimes preferred over the rigid ones for laryngeal examination. In the flexible case, we recommend head-mounted or stand-mounted microphones to be used instead of endoscope-mounted microphones for capturing the sound of voice, so that the microphone position is not dependent on the depth of the insertion of the endoscope into the nasal and vocal tract. Recommendations on the positioning of these microphone types with respect to the mouth were provided in other studies [7,8].

## 5. Practical Recommendations and Conclusions

The laryngoscope insertion depths determined here allow clinicians to specify the MTM distance in adults, avoiding the need to specifically measure this distance on patients during laryngoscopic examinations. It is applicable for microphones attached either to rigid laryngoscopes or to camera heads mounted on the rigid laryngoscopes.

To specify the MTM distance using our method, one should take the following steps:

1. Measure the position of the microphone with respect to the tip of the rigid endoscope (the distances *a* and *b*; Figure 1 and Figure 3);
2. Use the table value of the laryngoscope insertion depth, *D*_I_ (i.e., 9.7/9.4 cm for men, 8.9/8.7 cm for women, or 9.3/9.0 cm for all adults for 70°/90° endoscopes, respectively);
3. Calculate the typical MTM distance for that endoscope by using the Equation (2). These steps were used for specifying the MTM distances of the three microphones reported in Table 5 that can be considered typical case examples.

A single educated person or a technician could accomplish these tasks. It is recommended to mention the MTM distance when reporting the acoustic measurements of voice obtained during laryngoscopy. For instance, when the MTM distance was calculated to be 15 cm, and the SPL was found to be 80 dB(A), one could specify it as SPL@15 cm = 80 dB(A) [8].

**Figure A1:**
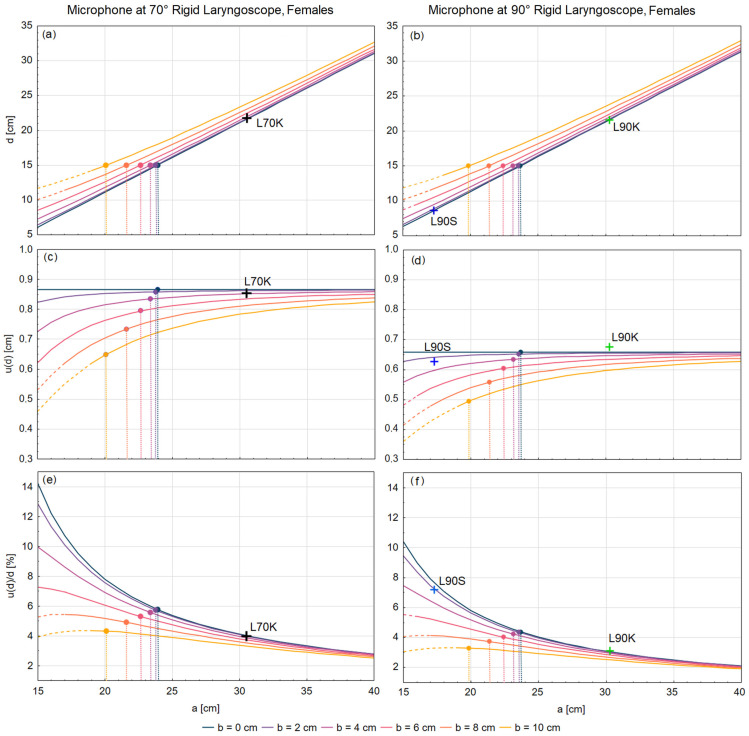
The average MTM distances, *d*, in females and their uncertainties *u*(*d*) for different microphone positions with respect to the tip of the laryngoscope (distances *a* and *b* indicated in Figure 1 and Figure 3). The results for the 70° laryngoscope are on the left (panels **a**,**c**,**e**) and for the 90° laryngoscope on the right (panels **b**,**d**,**f**). For the 90° laryngoscopes (**right** panels), the colored lines correspond to the pooled results from both the laryngoscopes, whereas the crosses indicate the results for the two laryngoscopes (L90K and L90S) separately. (**a**,**b**) MTM distance, *d*; (**c**,**d**) uncertainty of MTM distance, *u*(*d*); and (**e**,**f**) relative MTM distance uncertainty, *u*(*d*)/*d*. Crosses indicate results for the specific locations of the microphones used in this study. Dots indicate the standard MTM distance of 15 cm recommended by Wendler [9] for different values of *a* and *b*.

**Figure A2:**
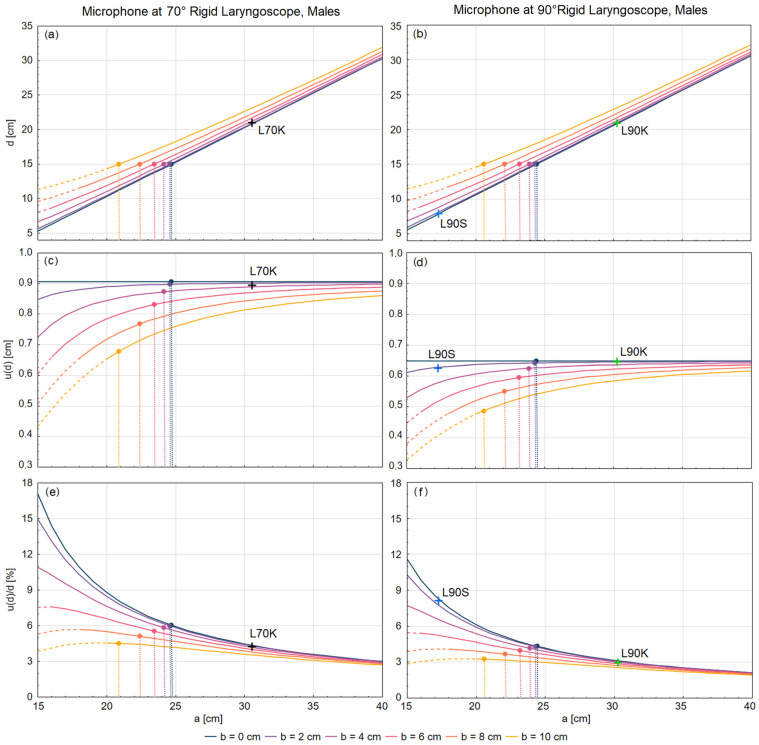
The average MTM distances, *d*, in males and their uncertainties *u*(*d*) for different microphone positions with respect to the tip of the laryngoscope (distances *a* and *b* indicated in Figure 1 and Figure 3). The results for the 70° laryngoscope are on the left (panels **a**,**c**,**e**) and for the 90° laryngoscope on the right (panels **b**,**d**,**f**). For the 90° laryngoscopes (**right** panels), the colored lines correspond to the pooled results from both laryngoscopes, whereas the crosses indicate the results for the two laryngoscopes (L90K and L90S) separately. (**a**,**b**) MTM distance, *d*; (**c**,**d**) uncertainty of MTM distance, *u*(*d*); and (**e**,**f**) relative MTM distance uncertainty, *u*(*d*)/*d*. Crosses indicate results for the specific locations of the microphones used in this study. Dots indicate the standard MTM distance of 15 cm recommended by Wendler [9] for different values of *a* and *b*.

One should be aware that the MTM distance obtained via this method is approximate; it will vary slightly among examined subjects. In our results, this is taken into account by specifying the distance uncertainty, calculated by using Equation (3). How much the MTM distance is expected to vary is specified in Figure 5c,d and in Figure A1c,d and Figure A2c,d in the Appendix A. The largest standard uncertainty of the MTM distance determined using our method is 0.91/0.75 cm for 70°/90° endoscopes, respectively (recall Figure 5c,d). This results in the relative uncertainty of ca. 3%/2.5% for microphones placed at the distance *a* = 40 cm from the tip of the endoscope, and it increases up to ca. 16%/12.5% when the *a* distance is decreased to 15 cm, for 70°/90° endoscopes, respectively (recall Figure 5e,f). Such uncertainties, especially those for longer *a* distances, are deemed acceptable. Importantly, the method offers the possibility of determining the MTM distance and its uncertainty already by the manufacturers of laryngoscopes. To achieve the standard laryngoscopic MTM distance of 15 cm recommended by Wendler [9], one should position the microphone at the *a* and *b* distances between 25 and 20 cm and between 0 and 10 cm, respectively, from the tip of the rigid laryngoscope (recall Figure 5 for the specific values). Specifying and reporting the MTM distance is relevant for properly interpreting the recorded voice and is helpful for better reproducibility and repeatability of laryngeal exams.

## Figures and Tables

**Table 1:** The division of the patients into three groups according to used laryngoscope types (L70K, L90K, and L90S). No statistically significant differences in age were found among the groups. SD = standard deviation.

Group	Number of Males (M), Females (F)	Type of Used Laryngoscope	Age (Mean ± SD)
1	20 M 20 F	L70K + L90S	M 53 ± 17
			F 42 ± 20
2	20 M 20 F	L90K + L90S	M 47 ± 17
			F 50 ± 18
3	20 M 20 F	L90S	M 50 ± 17
			F 44 ± 15

**Table 2:** Sizes of laryngoscopes used for laryngeal examination, the microphone positions with respect to the tip of the laryngoscope, and the distance of the center of the objective lens from the tip of the laryngoscope. VKG = videokymography.

Laryngoscope Code	Type of Examination	Angle (°)	Length of Tube, *l* (cm)	Reference Length, *r* (cm)	Microphone Distance, *a* (cm)	Microphone Distance, *b* (cm)	Lens to Tip Distance, *c* (cm)
L70K	VKG	70	18.64 ± 0.08	4.44 ± 0.02	30.52 ± 0.05	2.67 ± 0.06	0.46 ± 0.01
L90K	VKG	90	19.09 ± 0.06	4.31 ± 0.08	30.26 ± 0.04	2.67 ± 0.06	0.61 ± 0.01
L90S	Stroboscopy	90	17.27 ± 0.06	7.26 ± 0.05	17.27 ± 0.06	1.08 ± 0.03	0.30 ± 0.01

**Table 3:** The measured laryngoscope insertion depths for the three different laryngoscopes (L70K, L90K, and L90S) and for L90S pooled with L90K. The measurement results are listed as mean values and standard deviations (this holds for the other tables too). The results of the statistical tests for female–male differences are shown at the bottom of the table. Levels of significance were highlighted as *p*-value < 0.01 (**), and <0.001 (***).

	Sex	L70K		L90K		L90S		L90K + L90S	
		*n*	Insertion Depth, *D*_I_ (cm)	*n*	Insertion Depth, *D*_I_ (cm)	*n*	Insertion Depth, *D*_I_ (cm)	*n*	Insertion Depth, *D*_I_ (cm)
Insertion Depth	Females (F)	20	8.91 ± 0.86	20	8.84 ± 0.68	60	8.63 ± 0.63	80	8.68 ± 0.65
	Males (M)	20	9.70 ± 0.90	20	9.47 ± 0.63	60	9.39 ± 0.65	80	9.41 ± 0.64
	Adults (F + M)	40	9.31 ± 0.90	40	9.15 ± 0.72	120	9.01 ± 0.74	160	9.05 ± 0.74
F-test (*p*-value)	Females vs. Males	20/20	0.44	20/20	0.38	60/60	0.42	80/80	0.47
*t*-test (*p*-value)	Females vs. Males	20/20	0.009 **	20/20	0.005 **	60/60	<0.001 ***	80/80	<0.001 ***

**Table 4:** Laryngoscope insertion depth: differences among the different laryngoscopes—L70K, L90K, and L90S. Levels of significance were highlighted as *p*-value < 0.05 (*).

Sex	Insertion Depth Differences (cm)			Pair *t*-Test (*p*-Value)			F-Test (*p*-Value)		
	N	L70K–L90S	L90K–L90S	N	L70K vs. L90S	L90K vs. L90S	N	L70K vs. L90S	L90K vs. L90S
Females (F)	20/20	0.28 ± 0.56	0.04 ± 0.38	20/20	0.04 *	0.55	20/20	0.04 *	0.32
Males (M)	20/20	0.25 ± 0.76	0.05 ± 0.38	20/20	0.16	0.72	20/20	0.07	0.39
Adults (F + M)	40/40	0.27 ± 0.67	0.04 ± 0.38	40/40	0.17	0.80	40/40	0.04 *	0.47

**Table 5:** Mouth-to-microphone distances (*d*) and their uncertainties for the three laryngoscope-fixed microphones utilized in this study. The results were obtained by Formulas (1) and (2), using the microphone position characteristics *a* and *b* listed in Table 2 and the corresponding *D*_I_ values listed in Table 3.

Type of Laryngoscope	*d* Adults (cm)	*d* Females (cm)	*d* Males (cm)
L70K	21.38 ± 0.89	21.77 ± 0.86	20.99 ± 0.89
L90K	21.28 ± 0.72	21.59 ± 0.68	20.96 ± 0.63
L90S	8.33 ± 0.74	8.71 ± 0.63	7.95 ± 0.65

## Data Availability

The data presented in this study are available upon request from the corresponding author.
